# Spermaturia after Radical Prostatectomy: Is Surgical Preservation of Fertility Possible?

**DOI:** 10.1155/2013/124715

**Published:** 2013-04-07

**Authors:** Chad Reichard, Edmund S. Sabanegh, J. Stephen Jones, Khaled Fareed

**Affiliations:** Glickman Urological and Kidney Institute, Cleveland Clinic, 9500 Euclid Avenue, Q10-1, Cleveland, OH 44195, USA

## Abstract

Ease of sperm retrieval has not been previously described as a goal for patients undergoing radical prostatectomy for prostate cancer; however preservation of fertility is a known concern for some younger prostate cancer patients. We present the first known case of a patient with postejaculatory spermaturia following robotic assisted radical prostatectomy. We hypothesize that this is due to fistula formation between the vas deferens and the urinary tract.

## 1. Introduction

The mean age at diagnosis of prostate cancer from 2005 to 2009 was 67 years of age [1]. For the majority of patients in this age group, maintaining fertility is not a high priority. Cancer control, urinary continence, and erectile function are the more concerning outcomes for patients [2]. However, there are reports of younger men with prostate cancer undergoing sperm cryopreservation and percutaneous epididymal sperm extraction (PESA) for intracytoplasmic sperm injection (ICSI) [3–5]. Patients with low or intermediate risk disease and thus a more favorable prognosis of cure from surgery alone might benefit from an attempt at concomitant preservation of continuity of the vas with the urinary tract in order to facilitate ease of sperm retrieval for future efforts of conception.

## 2. Case Report

The patient was a 62-year-old man referred for urologic evaluation after new onset nocturia, frequency, and urgency with a PSA of 3.54 ng/mL. Four years prior to evaluation PSA was 2.38 ng/mL and eight years prior to it was 1.4 ng/mL. He denied hematuria or history of urolithiasis. Past medical history was significant for hypertension, hyperlipidemia, and coronary artery disease. IPSS was 22; Quality of Life Score was 3. SHIM was 21. He was married with two children. The patient subsequently underwent transrectal ultrasound guided (TRUS) biopsy of the prostate. Pathology revealed Gleason 3 + 3 prostate cancer in 1 of 20 cores with 5–10% of core involvement. The patient was counseled on the various treatment options for low risk, clinically localized prostate cancer, and elected to proceed with robotic assisted laparoscopic prostatectomy in September 2010. There was no clinically significant deviation from the usual operative technique; however, during posterior bladder neck division, the seminal vesicles and vasa deferentia were found to be particularly adherent to the rectal serosa and were taken in piecemeal fashion.

**Figure 1:**
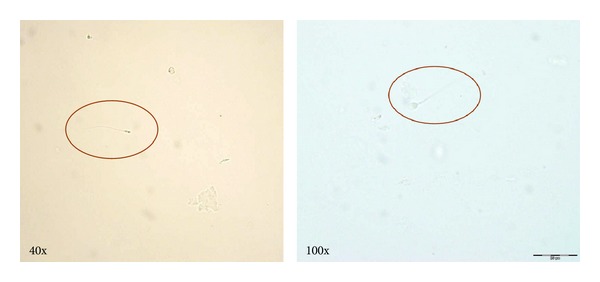
Postejaculatory urine microscopy.

Final pathology revealed pT2c, Gleason 3 + 3 prostatic adenocarcinoma with negative surgical margins. Seminal vesicles were present in the specimen and were negative for tumor. Two months postoperatively, he developed some right testicular discomfort and on exam the right epididymis was enlarged and tender. Microscopic urinalysis revealed 15–20 rbc/hpf, 5–10 wbc/hpf, no bacteria, and no evidence of sperm. Urine culture had insignificant growth. He was empirically treated with ciprofloxacin for epididymitis which resolved. At 18 months postoperatively, routine urinalysis was heme positive and microscopy revealed 1–3 nonmotile sperm per high powered field (hpf) (Figure 1). The patient denied any new urinary symptoms or other complaints. PSA remained undetectable. Subsequent postejaculatory urinalysis one week later revealed 10–12 nonmotile sperm/hpf.

## 3. Discussion

While data on fertility in cancer patients is prevalent in the literature, a Medline search using “fertility and prostatectomy” as well as more specific terms “spermaturia and prostatectomy” did not yield any results that described similar findings to the aforementioned case.

While this patient does not have motile sperm and thus does not have proven preservation of fertility, the fact that sperm are present in the postejaculation urine demonstrates a patent communication between the vas and the urinary tract. Because of the relative acidity of urine versus semen, it is not unexpected that the sperm is immotile in the urinary environment. Sperm motility might be preserved by concurrent urinary alkalinization as is performed for fertility preservation in the setting of neurogenic based retrograde ejaculation. Whether the presumed fistulous tract will remain patent, with continued passage of sperm, is unknown. In addition, there was no recent preoperative semen analysis; thus it is not known for sure whether oligoasthenospermia was present prior to surgery or is solely related to the surgically altered anatomy.

A review of a cryopreservation database by Williams IV et. al. demonstrated that with the exception of testicular cancer, men with most types of cancer have pretreatment semen parameters in the fertile range for density and in the intermediate range for motility. Six percent of the 717 semen samples from 409 men were from men with prostate cancer with a mean age at cryopreservation of 51.6 (range: 38.7–65.8) [5]. These data, in addition to the patient's prior fertility, make it unlikely that the oligoasthenospermia is solely due to preexisting pretesticular or testicular abnormality. Further followup of this patient is needed to see if sperm quality improves with time, or if azoospermia ensues. With the current data, it seems that any attempt to attain normospermia by purposeful incorporation of the vas in the vesicourethral anastomosis during prostatectomy represents a formidable technical challenge.

The morbidity of the outcome presented in this case is not clear. There may be a psychological detriment to informing patients who do not desire fertility that there are sperm in the postejaculatory urine even in spite of the fact that they are nonmotile and unlikely to cause pregnancy. This patient was monogamous with his postmenopausal wife, so he was not concerned that he could have even a remote possibility of fertility.

The current understanding of the pathophysiology of epididymo-orchitis in older men relates to seeding from urinary pathogens [6]. Thus there might be a theoretical increase in the risk of epididymo-orchitis in these patients since urine may more freely reflux into the vas, analogously to the phenomenon of urethroejaculatory duct reflux described in some children with epididymo-orchitis [7, 8]. The risk would most likely be lower in a patient that returns to normal voiding habits as this patient did, without stasis as a risk factor for urinary tract infection. However, this might be a greater consideration in patients that develop high pressure voiding, due to stricture of the bladder neck with associated retention, and stasis. The fact that this patient had mild epididymo-orchitis at 2 months postoperatively raises clinical suspicion; however, this was a single episode in two years of followup.

## 4. Conclusions

This case reports, to our knowledge, a previously undescribed finding of postejaculatory spermaturia in a patient status after robotic assisted laparoscopic radical prostatectomy. This finding is hypothesized to be due to formation of vasovesical fistula. We hypothesize that with future modifications of operative techniques, it may be possible to provide patients that desire to maintain fertility with a method to facilitate ease of sperm retrieval for artificial reproductive methods. However these techniques might be limited by high primary failure rates and potential added morbidity such as increased risk for epididymo-orchitis. In addition, it is unlikely success rates would be high enough to preclude the need for current methods of preoperative fertility preservation (i.e. sperm cryopreservation).

## References

[1] Howlader N, Noone AM, Krapcho M (2012). *SEER Cancer Statistics Review, 1975–2009 (Vintage 2009 Populations)*.

[2] Boyd BG, McCallum SW, Lewis RW, Terris MK (2006). Assessment of patient concern and adequacy of informed consent regarding infertility resulting from prostate cancer treatment. *Urology*.

[3] Bonetti TCS, Pasqualotto FF, Queiroz P, Iaconell A, Borges E (2009). Sperm banking for male cancer patients: social and semen profiles. *International Brazilian Journal of Urology*.

[4] Knoester PA, Leonard M, Wood DP, Schuster TG (2007). Fertility issues for men with newly diagnosed prostate cancer. *Urology*.

[5] Williams DH, Karpman E, Sander JC, Spiess PE, Pisters LL, Lipshultz LI (2009). Pretreatment Semen parameters in men with cancer. *Journal of Urology*.

[6] Wein (2012). Prostatitis and related conditions, orchitis, and epididymitis. *Campbell’s Urology*.

[7] Wiersma R (2009). Urethro-ejaculatory duct reflux in children: an updated review. *European Journal of Pediatric Surgery*.

[8] Raveenthiran V, Sam CJ (2011). Epididymo-orchitis complicating anorectal malformations: collective review of 41 cases. *Journal of Urology*.

